# Co-infection of *Borrelia burgdorferi* sensu lato and *Rickettsia* species in ticks and in an erythema migrans patient

**DOI:** 10.1186/1756-3305-6-347

**Published:** 2013-12-10

**Authors:** Ellen Tijsse-Klasen, Hein Sprong, Nenad Pandak

**Affiliations:** 1 Centre for Infectious Disease Control Netherlands, National Institute for Public Health and Environment (RIVM), Bilthoven, The Netherlands; 2 Department of Infectious Diseases, General Hospital “Dr. Josip Benčević”, Slavonski Brod, Croatia

**Keywords:** *Borrelia burgdorferi*, *Rickettsia monacensis*, *Rickettsia helvetica*, *Erythema migrans*, *Co-infection*

## Abstract

**Background:**

Lyme borreliosis is the most prevalent tick-borne disease in Europe. *Ixodes ricinus* also carries other pathogenic bacteria, but corresponding human diseases are rarely reported. Here, we compared the exposure to *Rickettsia helvetica* and *Rickettsia monacensis* with that to Lyme borreliosis spirochetes*.* We assumed that their exposure corresponds to their infection rate in questing *I. ricinus*.

**Findings:**

Three Rickettsia species were detected in ticks with a total prevalence of 7.9%, of which the majority was *R. helvetica* (78%) and *R. monacensis* (21%). From the same geographic area, skin biopsies of erythema migrans patients were investigated for possible co-infections with *Rickettsia* spp.. Forty-seven out of 67 skin biopsies were PCR positive for *Borrelia burgdorferi* s.l. and one sample was positive for *R. monacensis.* The Borrelia genospecies from the *R. monacensis* positive patient was identified as *Borrelia afzelii*. The patient did not show any symptoms associated with rickettsiosis.

**Conclusions:**

Co-infections of *I. ricinus* with *Rickettsia* spp. and *B. burgdorferi* s.l. were as high as expected from the individual prevalence of both pathogens. Co-infection rate in erythema migrans patients corresponded well with tick infection rates. To our knowledge, this is the first reported co-infection of *B. afzelii* and *R. monacensis.*

## Findings

Erythema migrans (EM) is an early sign of Lyme disease, which is caused by *Borrelia burgdorferi* sensu lato (s.l.) spirochetes that are transmitted by ticks. A typical EM is characterized by a bull’s-eye look with an expanding red ring of active infection and inner clearing but other presentations of EM can be found as well [1]. As a first sign of Lyme disease, it usually occurs at the site of the tick bite and is associated with tick attachment duration of more than 24 hours [2]. These premises make EM likely sites of co-infection with other tick borne pathogens that can be transmitted alongside *B. burgdorferi* s.l.. *Rickettsia* spp. are, next to *B. burgdorferi* s.l., the most common potential pathogens found in *Ixodes ricinus*, the main vector of Lyme disease in Europe [3-6]. Some *Rickettsia* species are well-established pathogens, while the pathogenic potential of others has not been fully elucidated [7]. Invasion and potentially infecting tissue at the tick-bite site is the first step tick-borne bacteria have to make in order to cause disease. We investigated the co-infection rates of ticks with these two pathogens and co-infections in EM patients from Croatia. Part of the data from this study has been published in a separate manuscript dealing with focus on the genetic variation of *Borrelia* genotypes [8].

For this purpose *I. ricinus* and EM skin biopsies were tested with a *B. burgdorferi* s.l. duplex qPCR and a conventional *Rickettsia* spp. PCR. DNA from vegetation ticks and skin biopsies of EM patients was extracted and tested for the presence of *B. burgdorferi* s.l. DNA [8]. Samples were tested for *Rickettsia* by conventional PCR on the 16S rRNA gene and PCR-positive samples were sequenced and analyzed as described [4,9]. Positive and negative controls were run with each tested batch. Positive control for the *Rickettsia* spp. PCR was a *Rickettsia africae* positive patient sample [10]. *Rickettsia africae* is not found in *I. ricinus* and can be differentiated from rickettsial species in *I. ricinus* based on the 16S rRNA gene.

### Prevalence of *Borrelia burgdorferi* s.l. and *Rickettsia* spp. in Croatian ticks and EM skin biopsies

Of 1432 *I. ricinus* tested for *Borrelia* 254 (17.7%) were positive. Of 1273 *I. ricinus* tested for *Rickettsia* spp. 101 (7.9%) were positive [8]. Of these, 79 (78%) were identified as *Rickettsia helvetica* (100% homology to L36212)*,* 21 (21%) as *Rickettsia monacensis* (100% homology to [GenBank:DQ100164]) and one (1%) as *Rickettsia raoultii* (100% homology to [GenBank:DQ365809]). Of 67 EM skin samples 47 were *B. burgdorferi* s.l. positive (70%) and one (1.5%) was *Rickettsia* sp. positive. The latter sample was also positive for *B. afzelii* and the rickettsial species was identified as *R. monacensis* (100% homology to [GenBank:DQ365809]).

### Case description

The *R. monacensis* positive patient was an eight-year-old girl living in an urban environment. Six days prior to the hospital visit the patients mother had noticed a red annular rash on her daughter’s face. The rash expanded throughout the following day and during the hospital visit three EM lesions were noticed: around the left eye (7×14 cm), at the anterior side of the right thigh (9×12 cm) and on the right gluteus (11×17 cm). The skin biopsy was taken from the gluteal EM. The patient complained about itching but no other symptoms were reported or noted during the visit. Neither the patient nor her mother recollected any tick bites. After the diagnosis of Lyme borreliosis presenting as multiple EMs following dissemination of the infection, treatment with azithromycin (10 mg/kg) was initiated. The antibiotic was administered twice on the first treatment day and once daily until day five. The EMs started to fade during the second treatment day and had disappeared by day four. Serology later confirmed the diagnosis of Lyme borreliosis but did not show a seroconversion towards spotted fever group Rickettsia (data not shown). Throughout the disease course, no symptoms indicating a rickettsial co-infection were noticed. At check-ups two weeks and two months after the initial visit, the skin was clear and the patient was fully recovered.

## Discussion

Derived from the *Borrelia* spp. and *Rickettsia* spp. infection rates in ticks of 17.7% and 7.9%, respectively, a theoretical co-infection rate of 1.4% was calculated (infection rate of *Borrelia* spp. x infection rate of *Rickettsia* spp.). The measured co-infection rate in the 1273 *I. ricinus* that were tested for both bacteria was 1.4%. The theoretical and actual co-infection rates in ticks were also calculated for the combinations of the commonest *Borrelia* genospecies (*Borrelia garinii* and *B. afzelii*) and *Rickettsia* species (*R. helvetica* and *R. monacensis*) and theoretical and actual co-infection rates for these combinations were also nearly identical (data not shown). The detection of *R. raoultii* in *I. ricinus* is surprising as this rickettsial species is associated with *Dermacentor* spp. The *R. raoultii* prevalence in this study was only 0.08% and detection of this DNA might be attributed to a recent infected blood meal or a contamination of the tick sample with material from *Dermacentor marginatus* ticks, which had been collected simultaneously.

The measured co-infection rate of 1.4% in ticks indicates that this percentage of EM patients has also been exposed to *Rickettsia* spp*.*. In our study population of 67 EM cases, this would translate to one patient (calculated: 0.94 patients). Indeed one skin sample was found *Rickettsia* spp. positive. Although the study population is limited, this indicates that transmission of *Rickettsia* spp. can occur alongside of *Borrelia* spp. if the tick carries a double infection. The current study population was too small to draw a definite conclusion on the virulence and pathogenicity differences of different rickettsial species found in *I. ricinus*. However, as *R. helvetica* was almost four times more common in ticks than *R. monacensis*, by chance, *R. helvetica* would have been more likely to cause co-infections in EM. Both, *R. helvetica* and *R. monacensis*, are thought to be pathogenic but the full clinical picture or the virulence are not known for either of these two species [11,12]. In the current case, the patient did not seem to suffer any symptoms associated with a *Rickettsia* spp. infection. The erythematous lesions were attributed to the *B. burgdorferi* s.l. infection and the patient recovered completely after a five-day course of antibiotics. Average incubation time in children with multiple EM has been reported to lie between 10 and 25 days [13,14]. The rickettsial bacteria in the skin lesions have thus survived in the skin for a similarly long period without being eliminated by the patient’s immune system. Future case reports and epidemiological studies would be necessary to evaluate the clinical spectra and virulence of *R. helvetica* and *R. monacenis*.

## Conclusions

In conclusion, the co-infection rates in EM patients with *Rickettsia* spp. was as high as predicted by tick co-infection rates. All symptoms of the patient with *R. monacensis* co-infection can be explained by the *B. afzelii* infection and the virulence of this rickettsial agent remains unclear. Nevertheless, this study highlights the importance of considering co-infections when treating Lyme borreliosis.

## Consent

Informed written consent for publication was obtained from the parents of the patient.

## Competing interests

The authors declare that they have no competing interests.

## Authors’ contributions

NP and HS designed the study and NP collected samples and patient data. ETK carried out laboratory experiments and conducted data analysis. All authors contributed to the manuscript and approved the final version of the manuscript.
